# Supplementary material for: Impact of climate change on maternal health outcomes: An evidence gap map review
Source: PLOS Glob Public Health. 2024 Aug 19;4(8):e0003540. doi: 10.1371/journal.pgph.0003540 (PMC11332935; doi:10.1371/journal.pgph.0003540)
Supplement: S1 File — (DOCX) [file pgph.0003540.s001.docx]

Campbell Collaboration checklist for evidence and gap maps: Reporting standards

Developed by Howard White, Vivian Welch, Terri Pigott, Zack Marshall, Birte Snilstveit, Christine Mathew and Julia Littell

DRAFT Version 1.2 (Updated February 2018)

Note for authors: This document provides detailed methodological expectations for the reporting of Campbell Collaboration evidence and gap maps (EGMs).

Status:

Mandatory means that a new EGM will not be published if this standard is not met.

Highly desirable means that this should generally be done but that there are justifiable exceptions. There may be legitimate variation between or within Campbell Coordinating Groups in the relative emphasis placed on compliance with highly desirable standards. The emphasis placed on compliance with highly desirable standards will remain at the discretion of each Campbell Coordinating Group.

Optional means this is done at the authors’ discretion.

M = Presentation of the map

R = Accompanying descriptive report.

| **Item No.** | **Status** | **Item Name** | **Standard** | **Rationale and elaboration** | **Reference to Checklist Item No.** | **Page#** |
| --- | --- | --- | --- | --- | --- | --- |
| **Title and authors** | | | | |  |  |
| ER1 | Highly desirable  (M,R) | Format of title | Follow the standard Campbell EGM title template. | The title captures the scope of the map with the format: {Intervention or problem for} and {Population} or {location/situation} or {outcome}: An Evidence and Gap Map {of study type/design}.  Note: {…} indicates optional |  | 1 |
| ER2 | Mandatory  (R) | Authors | List names and affiliations of all authors | All authors should be named, with their affiliation. List contact details of the lead author. |  | 1 |
| **Abstract** | | | | |  |  |
| ER3 | Mandatory  (R) | Writing the abstract | Prepare a structured abstract to provide a succinct overview of the EGM. In the interests of brevity it is highly desirable for authors to provide an abstract of less than 700 words, and it should be no more than 1000 words in length. | Abstracts are a prominent, publicly accessible summary of the EGM. They should convey key information about the EGM including scope and overview of the evidence. |  | 2 |
| ER4 | Mandatory  (R) | Abstract, Background | Summarize the rationale and scope of the EGM. | The scope should be provided along with the main intended use and intended users of the EGM. Indicate whether a stakeholder or advisory group was engaged. |  | 2 |
| ER5 | Mandatory  (R) | Abstract, Objectives | State the main objective(s), preferably in a single concise sentence. | The objective(s) should be expressed in terms that relate to the intervention/problem, location, population, outcome and/or study design. |  | 2 |
| ER6 | Mandatory  (R) | Abstract, Search methods | Provide the date of the last search from which records were evaluated and any studies identified were incorporated into the EGM, and an indication of the databases and other sources searched. | Abstracts should aim to give readers brief but key information about the comprehensiveness of the search and the currency of the information in the map.  The abstract must include the month and year of the set of searches up to which items in the map have been identified. This date should reflect the date of the most recent set of searches from which all records have been screened for relevance and any studies meeting the eligibility criteria have been fully incorporated into the map.  The amount of information regarding the search should be indicative of the process rather than provide specific details. In the interests of brevity certain details regarding the overall process may need to be moved to the full text of the report. |  | 2 |
| ER7 | Mandatory  (R) | Abstract, Selection criteria | Summarize eligibility criteria of the evidence and gap map, including information on types of evidence, interventions, population, outcomes, and setting, as appropriate. | Any extensions to eligibility criteria to address adverse effects, economic issues, or qualitative research should be mentioned. |  | 2 |
| ER8 | Mandatory  (R) | Abstract, Data collection and analysis | Summarize any noteworthy methods for identifying studies, collecting data, and evaluating risk of bias/study quality. | This section of the abstract should indicate the rigor of the methods that underpin the results reported subsequently in the abstract. It does not need to replicate detailed description of the methods in the main text of the EGM report.  Details of how many people were involved in the screening process and collection of information about any included studies are not necessary in the abstract.  The abstract should prioritize the disclosure of non-standard approaches. For example, rather than disclosing all domains applied in the assessment of risk of bias/study quality, notable variations on the standard approach should be given, such as non-standard tools that were used. |  | 2 |
| ER9 | Mandatory  (R) | Abstract, Main results: number of studies | Report the number of included studies, reporting systematic reviews and primary studies separately. | The total number of included studies should be stated. The number of systematic reviews and primary studies should be reported separately.  It may be appropriate to provide numbers of studies for specific population groups, interventions, outcomes, or settings. |  | 2 |
| ER10 | Highly desirable  (R) | Abstract, Main results: study characteristics | Provide a brief description of the coverage of evidence by key dimensions to provide an overview of the body of evidence (e.g. region, and intervention type). Identify clusters of evidence and evidence gaps. | Summarizing the study characteristics, and identifying main evidence and gaps, will provide readers of the abstract with the main findings. |  | 2 |
| ER11 | Highly desirable | Abstract, Main results: risk of bias, study quality or confidence of included studies | Provide a comment on the risk of bias/quality assessments/confidence of the evidence. | The risk of bias/study quality/confidence assessments are a key finding and form a fundamental part of the strength of the evidence base. If risks of bias/study quality/confidence differ substantially for different interventions and outcomes, this may need to be mentioned. |  | 2 |
| ER12 | Mandatory | Abstract, Implications for research | State key conclusions drawn. | Authors’ conclusions may include both implications for practice and implications for research. |  | 2 |
| ER13 | Mandatory | Completeness of main report text | Ensure that all findings reported in the abstract also appear in the main text of the EGM report. | It is important that all statements in the abstract are supported by analysis in the main report. |  | 2 |
| ER14 | Mandatory | Consistency of summary versions of the EGM | Ensure that reporting of objectives, important outcomes, caveats and conclusions is consistent between the abstract and the main report. | Summary versions of the EGM should be written on the assumption that they are likely to be read in isolation from the rest of the map. |  | 2 |
| **Background section of EGM report** | | | | |  |  |
| ER15 | Mandatory | Background | Provide a concise description of the scope of the EGM, and why it is important to do the EGM. | EGMs should have a clearly defined and well-reasoned rationale. Outlining the context of the scope of the EGM is useful to readers and helps to establish the motivation for the map. | EP1 | 3 |
| ER16 | Mandatory | Background references | Support all key supporting statements with references. | Claims or statements regarding aspects such as prevalence and mechanisms of action should be substantiated and, where available, supported by external evidence. |  | 3 |
| ER17 | Mandatory | Background text | Do not use plagiarized text. | Unacknowledged copying from the work of other people is not acceptable. There may however be situations in which the same text appears in different reports, for example when the reports are prepared by the same team.  Content that is identical to, drawn or copied from standard texts may be acceptable but must be referenced. Ensure any verbatim quotations of more than a few words are shown in quotation marks and clearly acknowledge (i.e., cite) all sources. |  | 3,4 |
| ER18 | Mandatory | Objectives | State the objectives, where appropriate in a single concise sentence. | The primary objective of a Campbell EGM should be to provide an overview of the evidence pertaining to the scope of the map. The objective should be expressed in terms of relevant dimensions that can relate to the problem/issue, population, setting, intervention, or outcomes of interest. | EP2 | 4,5 |
| ER19 | Mandatory | Types of evidence | State the types of evidence being shown in the EGM explicitly in the objectives | The primary aim of a Campbell EGM is to present all available quality evidence related to the scope for that EGM in a user-friendly format. | EP11  EP12 | 6 |

| **Methods** | | | | |  |  |
| --- | --- | --- | --- | --- | --- | --- |
| ER20 | Mandatory  (R) | Reference protocol | Cite the protocol for the EGM. | The reader should be made aware that the EGM is based on a published protocol. The protocol should be cited using the last publication citation for the protocol in the Campbell Library. |  | 5 |
| **Criteria for inclusion and exclusion of studies in the EGM** | | | | |  |  |
| ER21 | Mandatory  (R) | Eligibility criteria for types of study: types of evidence | State eligible types of evidence, and provide a justification for the choice. | Different types of evidence may be shown in an EGM. While EGMs typically present evidence of effectiveness, they can include both quantitative and qualitative evidence. EGMs should specify which of the outcomes will be addressed using each type of evidence. | EP12 | 6 |
| ER22 | Mandatory  (R) | Eligibility criteria for types of study: study report status | Campbell EGM should include all relevant evidence regardless of publication status and language of publication. Any exceptions should be explicitly stated and justified. | Studies should be included irrespective of their publication status, and electronic availability. If studies are excluded based on their language of publication, explicit justification for this exclusion should be provided. | EP14 | 6 |
| ER23 | Mandatory  (R) | Eligibility criteria for types of participants | State eligibility criteria for participants, including any criteria around location, setting, status, or definition of condition and demographic factors, and how studies including subsets of relevant participants are handled. | Any notable restrictions on the eligibility criteria of the map should be given and explained (e.g., exclusion of people under or over a certain age, specific settings of intervention). | EP4  EP5 | 6 |
| ER24 | Mandatory  (R) | Eligibility criteria for types of interventions | State eligibility criteria for interventions and comparators, including any criteria around delivery, dose, duration, intensity, co-interventions, and characteristics of complex interventions. | Define in advance the eligible interventions and the interventions against which these can be compared in the included studies if applicable | EP7  EP8 | 6 |
| ER25 | Mandatory  (R) | Role of outcomes | State whether outcomes are used as eligibility. If so, define. | Studies should never be excluded from an EGM solely because no outcomes of interest are *reported*. However, on occasion it will be appropriate to include only studies that *measured* particular outcomes. | EP9 | 6 |
| ER26 | Mandatory  (R) | Outcomes of interest | State outcomes of interest to the EGM, and define acceptable ways of measuring them. | Explain how multiple variants of outcome measures (e.g., definitions, assessors, scales, and time points) are addressed, indicating which fall under a single outcome title in the EGM. | EP17 | 20 |

| **Search methods for identification of studies** | | | | |  |  |
| --- | --- | --- | --- | --- | --- | --- |
| ER27 | Mandatory  (R) | Search sources | List all sources searched, including: databases, trials registers, web sites, and grey literature. Database names should include platform/provider name and dates of coverage; web sites should include full name and URL. State whether reference lists were searched and whether individuals or organizations were contacted. Indicate whether stakeholders were consulted. | The search sources should be cited in sufficient detail for readers to be able to replicate the search results. | EP18-23 | 6 |
| ER28 | Mandatory  (R) | Latest searches | Provide the date of the last search and the issue/version number (where relevant) for each database whose results were evaluated and incorporated into the EGM. If a search was re-run prior to publication, the results of which were not incorporated, explain how the results were dealt with and provide the date. | The EGM report should provide the search date from which studies have been retrieved and assessed for inclusion. This is the date up to which the EGM is valid. It should reflect the date of the most recent set of searches from which all records have been screened for relevance and any studies meeting the eligibility criteria have been fully incorporated into the EGM (studies may be awaiting classification if, for example, the EGM authors are awaiting translation or clarification from authors or sponsors).  Since the map is likely to have drawn on searches conducted across multiple databases, it is possible that searches were performed on more than one date. The earliest date of the most recent set of searches should be provided in the map report and as the hard-coded date of the last search. The remaining dates for other databases should be reported in an appendix.  If a ‘catch-up’ search was run subsequent to the EGM being written up, any relevant studies not yet assessed for inclusion should be listed an annex to the report entitled ‘Studies awaiting assessment’. |  | 6 |
| ER29 | Mandatory  (R) | Search restrictions | Specify and justify any restrictions placed on the time period covered by the search. | Justify the use of any restrictions in the search strategy on publication date, publication format or language. | EP24 | 6 |
| ER30 | Mandatory  (R) | Searches for different types of evidence | If the EGM has specific eligibility criteria to include additional studies such as studies of adverse effects, economics evidence or qualitative research evidence, describe search methods for identifying such studies. | EGMs may extend beyond a focus on the effects of interventions and address specific additional types of evidence. | EP19 | 6 |
| ER31 | Mandatory  (R) | Search strategies for bibliographic databases | Present the exact search strategy (or strategies) used for each database in an Appendix, including any limits and filters used, so that it could be replicated. | Search strategies that are available elsewhere (e.g., standard methodological filters, or strategies used to populate a specialized register) may be referenced rather than reproduced. Including numbers of hits for each line in the strategy is optional. | EP22 | 5,6 |
| ER32 | Highly desirable  (R) | Search strategies for other sources | Report the search terms used to search any sources other than bibliographic databases (e.g., trials registers, the web, direct contact with primary study authors), and the dates of the searches. | Some of this information might be best placed in an Appendix. |  | 5,6 |
| **Data collection and analysis** | | | | |  |  |
| ER33 | Mandatory  (R) | Inclusion decisions | State how inclusion decisions were made (i.e. from search results to included studies), clarifying how many people were involved and whether they worked independently. | There should be at least two people working independently to determine whether each study meets the eligibility criteria, and define in advance the process for resolving disagreements. | *EP26* | *6,7* |
| ER34 | Mandatory  (R) | Data collection process | State how data were extracted from reports of included studies, clarifying how many people were involved (and whether independently), and how disagreements were handled. Describe data collection process for any reports requiring translation. | The data extraction form should be piloted. It is highly desirable to use (at least) two people working independently to extract study characteristics from reports of each study, and define in advance the process for resolving disagreements. | EP30 | 6,7 |
| ER35 | Highly desirable  (R) | Requests for data | Describe attempts to obtain or clarify data from individuals or organizations. | Report how the team sought key unpublished information that is missing from reports of included studies. | ER23 | 6 |
| ER36 | Mandatory  (R) | Data items | List the types of information that were sought from reports of included studies. | Collect characteristics of the included studies in sufficient detail to construct the map and populate final tables in the report. |  | *7* |
| ER37 | Mandatory (R) | Tools to assess risk of bias/study quality/confidence in individual studies | State the tool(s) or coding strategies used to assess the primary study quality/risk of bias/confidence for included studies, how the tool(s) or coding strategies were implemented, and the criteria used to assign studies, for example, to judgments of low risk, high risk, and unclear risk of bias; low quality or high quality. | It is highly recommended to assess the study quality or risk of bias or confidence for each included study. For randomized trials, the Cochrane 'Risk of bias' tool might be used, involving judgments and supports for those judgments across a series of domains of bias. | EP25 | 7 |

| Results | | | | |  |  |
| --- | --- | --- | --- | --- | --- | --- |
| Description of studies | | | | |  |  |
| ER38 | Mandatory  (R) | Flow of studies  (R) | Provide information on the flow of studies from the number(s) of references identified in the search to the number of studies included in the EGM, ideally using a flow chart. Clarify how multiple references for the same study relate to the individual studies. | Document the selection process in sufficient detail to complete a PRISMA flow chart and a table of ‘Characteristics of excluded studies’.  Collate multiple reports of the same study, so that each study rather than each report is the unit reported in the EGM. |  | Page#6 Fig.2, |
| ER39 | Mandatory  (R) | Excluded studies  (R) | List in the report key excluded studies (i.e., those a reader might reasonably have expected to find) and provide justification for each exclusion. | The table of ‘Characteristics of excluded studies’ is intended as an aid to users rather than a comprehensive list of studies that were identified but not included. List here any studies that a user might reasonably expect to find in the EGM to explain why it is excluded. |  | Page#6 Fig.2 |
| See *Handbook* 7.2.5. | | | | |  |  |
| ER40 | Highly desirable | Studies awaiting classification  (R) | List in the report the characteristics of any studies that have been identified as potentially eligible but have not been incorporated into the map. | Users of the EGM will be interested to learn of any potentially relevant studies that have been conducted which are known to the map team but have not yet been incorporated in to the EGM. This will help them to assess the stability of the map findings. These should be listed in the table of ‘Characteristics of studies awaiting classification’, along with any details that are known. |  | Page#6 Fig.2 |
| ER41 | Mandatory | Provide details of references  (M) | In the map provide a link to full reference for each included study, including multiple citations if applicable | The usefulness of the map to the user is greatly enhanced if full details of the included studies are readily accessible from the map. |  | 9,10 & Figure 1, |
| ER42 | Highly desirable | Included studies  (M) | Provide a brief structured abstract of all included studies. This should include the characteristics of the study design, objectives and dimensions of the map. | The structured abstract should include publication details, study design, objective, sample dimensions from the EGM framework and main findings. |  | 9,10 Figure 1, |
| ER43 | Highly desirable | Filter for selected characteristics of included studies | Provide a filter for included studies to enable a user of the EGM to assess the availability of evidence for their own setting (e.g. region, study date, population characteristics) | Users often want to view a subset of the evidence, such as for a particular region. Adding filters which can be applied for this purpose enhances the usefulness of the map. |  | 9,10, Figure 1, |
| **Study quality/risk of bias in included studies** | | | | |  |  |
| ER 44 | Mandatory | Quality or confidence of systematic reviews | All systematic reviews should be appraised for quality or confidence. |  | EP27 | 7, Supplementary file, 3 |
| ER45 | Highly desirable  (M) | ‘Risk of bias’, study quality or confidence coding | Use a coding (colours or shapes) to indicate the risk of bias, study quality or confidence for each included study, with judgments about risks of bias. | Assess the risk of bias, study quality or confidence for each included study. Display in the map. | EP27 | 7, Supplementary file, 3 |
| ER46 | Highly desirable  (R) | ‘Risk of bias’, study or confidence table | Present a “risk of bias and/or study quality or confidence” table for each included study, with judgments about risks of bias, and explicit supports for these judgments. | A summary table should be provided overviewing the risk of bias, study quality or confidence of the studies. | EP27 | 7, Supplementary file, 3 |
| ER47 | Mandatory  (R) | Summarize quality of systematic reviews | Provide a brief narrative summary of the quality or confidence of systematic reviews in results. | It may be helpful to identify any systematic reviews considered to be at low risk of bias for particular key outcomes or with high quality for specific methodological characteristics. |  | 7, Supplementary file, 3 |
| ER48 | Highly desirable  (R) | Summary assessments of risk of bias/study quality | Summarize the study quality/risk of bias by dimensions of the map. | It is helpful to users to know the quality of evidence as it relates to specific map dimensions (e.g. across intervention and outcome categories). This information can help inform research funding decisions. |  | 7, Supplementary file, 3 |
| **Effects of interventions** | | | | |  |  |
| ER49 | Highly desirable  (R) | Number of Tables and Figures | Restrict the number of Tables and Figures to a small number (six or less) to convey key findings without affecting the readability of the EGM report text. | Tables and Figures may be added to EGM report and included in the body of the text. Reports should try to avoid including a large number of Tables and Figure. Additional or supplementary Tables and Figures can be included as appendices, as appropriate. |  | 8,9,10 |
| ER50 | Mandatory  (R) | Consistency of reporting | Ensure that all evidence reported in the map is captured in the EGM report, and that the report text is consistent with all Tables and Figures  . | The iterative nature of EGM production may mean that inconsistencies emerge between text and the corresponding tables and figures. These should all be checked to ensure such inconsistencies are eliminated. |  | 8,9,10 |
| ER51 | Optional  (R) | Assessments of the quality of the body of evidence | Provide justification or rationale for any measures of the quality of the body of evidence for each key outcome. | Justify and document all assessments of the quality of the body of evidence (for example downgrading or upgrading if using the GRADE tool).) |  | 8,9,10 |
| **Discussion** | | | | |  |  |
| ER52 | Highly desirable  (R) | Discussion headings | Include in the report the standard headings when writing the Discussion. | Five standard headings are recommended (‘Summary of main results’, ‘Overall completeness and applicability of evidence’, ‘Quality of the evidence’, ‘Potential biases in the mapping process. |  | 10 |
| ER53 | Mandatory  (R) | Limitations | In the report, discuss limitations of the map (e.g., incomplete identification of studies, reporting bias), and the implications of any study-level or outcome-level risk of bias/quality/ confidence assessments of the evidence in the map. | EGM authors must explicitly state the limitations of the map. These limitations should be addressed in the discussion headings of ‘Quality of the evidence’ and ‘Potential biases in the mapping process.’ If those two headings are not used, then at minimum, the map report should include a ‘Limitations’ heading that addresses all potential limitations. |  | 13 |
| **Authors’ conclusions** | | | | |  |  |
| ER54 | Mandatory  (R) | Conclusions: implications for research | If recommending further research, structure the implications for research to address the nature of evidence required, including population, intervention comparison, outcome, and type of study. | Researchers and research funders are an important user group of Campbell EGMs. Recommendations for future research should offer constructive guidance on addressing the remaining uncertainties identified by the map. This is particularly important for maps that identify few or no studies. |  | 12,14 |
| **Acknowledgements** | | | | |  |  |
| ER55 | Mandatory  (R) | Acknowledgements | Acknowledge the contribution of people not listed as authors of the map, including any assistance from Campbell Coordinating Groups, non-author contributions to searching, data collection, study appraisal or statistical analysis, and the role of any funders. | All contributors should be identified by name and affiliation, with their contribution indicated. |  | 14 |
| **Contributions of authors** | | | | |  |  |
| ER56 | Mandatory  (R) | Contributions of authors | Describe the contributions of each author. | The contribution of each author to the map should be listed. |  | 14,15 |
| **Declarations of interest** | | | | |  |  |
| ER57 | Mandatory  (R) | Declarations of interests | Report any present or past affiliations or other involvement in any organization or entity with an interest in the map’s findings that might lead to a real or perceived conflict of interest. | The nature and extent of the affiliation or involvement (whether financial or non-financial) should be described. An additional consideration for authors of EGMs is the declaration of involvement in studies that were included in the map. It is important to note that authors who were involved in primary studies must not be involved in the data extraction/coding/critical appraisal of those studies. |  | 14 |
| **Differences between protocol and map** | | | | |  |  |
| ER58 | Mandatory  (R) | Changes from the protocol | Explain and justify any changes from the protocol (including any *post hoc* decisions about eligibility criteria. | Justify any changes to eligibility criteria or outcomes studied. In particular, post hoc decisions about inclusion or exclusion of studies should keep faith with the objectives of the map rather than with arbitrary rules. |  | *NA* |
| **Sources of support** | | | | |  |  |
| ER59 | Mandatory  (R) | Sources of support | List sources of financial and non-financial support for the map and the role of the funder, if any. | All sources of support should be listed. |  | 14 |
