# Supplementary material for: Impact of climate change on maternal health outcomes: An evidence gap map review
Source: PLOS Glob Public Health. 2024 Aug 19;4(8):e0003540. doi: 10.1371/journal.pgph.0003540 (PMC11332935; doi:10.1371/journal.pgph.0003540)
Supplement: S3 File — (DOCX) [file pgph.0003540.s003.docx]

**S3 File. Search strategy: climate change and maternal health outcomes**

| **Ovid MEDLINE(R) ALL <1946 to January 13, 2024>** |  |  |  |
| --- | --- | --- | --- |
|  |  |  |  |
| 1 |  | Maternal Health/ | 2282 |
| 2 |  | Maternal Mortality/ or Perinatal Death/ | 13316 |
| 3 |  | exp maternal health services/ or exp perinatal care/ | 57701 |
| 4 |  | exp pregnancy/ or exp labor, obstetric/ or exp parturition/ or exp pregnancy outcome/ or exp pregnancy, multiple/ | 1002543 |
| 5 |  | exp pregnancy complications/ or exp abortion, spontaneous/ or exp fetal death/ or exp fetal diseases/ or exp hypertension, pregnancy-induced/ or exp obstetric labor complications/ or exp pregnancy complications, cardiovascular/ or exp pregnancy complications, infectious/ or exp pregnancy complications, neoplastic/ or exp trophoblastic neoplasms/ or exp prenatal injuries/ or exp puerperal disorders/ or exp lactation disorders/ or exp mastitis/ | 473037 |
| 6 |  | (matern* adj3 (health* or wellbeing or well-being or wellness or mortality or death* or disease* or infect* or condition* or stress*)).mp. | 82317 |
| 7 |  | ("pregnancy complication*" or "pregnancy outcome*" or "birth complicat*" or "birth* outcome*").mp. | 236111 |
| 8 |  | ((prenatal or antenatal or postnatal or perinatal or antepartum or postpartum or obstetric* or pregnan*) adj3 (care or service* or check-up* or checkup* or health* or visit*)).mp. | 99698 |
| 9 |  | ((pregnan* or gestation*) and (hypertensi* or "high blood pressure*" or "elevated blood pressure*")).mp. | 41104 |
| 10 |  | (eclamp* or preeclamp* or pre-eclamp*).mp. | 55577 |
| 11 |  | mothers/ | 55419 |
| 12 |  | (pregnan* or mother* or childbearing).mp. | 1288947 |
| 13 |  | or/1-12 | 1356726 |
| 14 |  | carbon cycle/ or carbon sequestration/ or climate change/ or global warming/ or droughts/ or El Nino-Southern Oscillation/ or floods/ or greenhouse effect/ or Hot Temperature/ or ("Arctic amplification" or "Arctic shrinkage" or "carbon footprint" or "carbon offset*" or "carbon sequestration" or "carbon sink" or "carbon sinks" or (chlorofluorocarbon* adj2 (release or concentration* or atmosphere*)) or "climate change*" or deglaciation or desertification or "natural disaster*" or drought or (earth adj2 warming) or El Nino or La Nina or "emissions reduc*" or "emissions trading" or flood* or ((glacial or galcier) adj3 (retreat* or melt*)) or "global radiation" or "global temperature*" or "global warming" or "greenhouse effect" or "greenhouse gas" or "heat wave*" or hurricane* or "ice cap melt*" or "ice mass loss*" or "Kyoto Protocol" or "landslide*" or "land slide*" or "mudslide*" or "mud slide*" or (ozone adj2 hole) or "Paris Accord" or "permafrost melt*" or "polar amplification" or "polar ice melt*" or "sea ice shrinkage" or "sea level rise*" or "sea surface warming" or "storm surge*" or "thermohaline circulation" or ((extreme or severe or destructive) adj2 (temperature* or weather* or storm* or hail or wind* or heat)) or tornado* or typhoon* or wildfire* or "wild fire*").mp. | 283007 |
| 15 |  | Heat-Shock Response/ | 9256 |
| 16 |  | (heat adj2 (stress* or shock* or cramp*)).mp. | 97226 |
| 17 |  | 14 or 15 or 16 | 363955 |
| 18 |  | 13 and 17 | 5333 |
| 19 |  | exp animals/ not exp humans/ | 5126093 |
| 20 |  | (pollen* or plant* or flora or soil* or agricultur* or "organic matter" or chlorophyll* or crop? or seedling* or seed? or cell?).ti. | 2467849 |
| 21 |  | (pig? or rat? or animal* or mouse or mice or cow? or poultry).ti. | 1854705 |
| 22 |  | 19 or 20 or 21 | 7385208 |
| 23 |  | 18 not 22 | 3007 |
| 24 |  | limit 23 to (english language and yr="2013 -Current") | 1435 |

| **Embase <1974 to 2024 January 13>** |  |  |
| --- | --- | --- |
|  |  |  |
| 1 | maternal welfare/ | 17291 |
| 2 | maternal mortality/ | 24160 |
| 3 | exp perinatal mortality/ | 29395 |
| 4 | exp fetus death/ or perinatal death/ | 49485 |
| 5 | maternal health service/ | 2840 |
| 6 | perinatal care/ or exp maternal care/ | 69952 |
| 7 | exp pregnancy/ | 773341 |
| 8 | childbirth/ or labor/ or labor support/ or term birth/ | 55552 |
| 9 | parameters concerning the fetus, newborn and pregnancy/ or fetus outcome/ or pregnancy outcome/ or prenatal mortality/ | 92540 |
| 10 | exp pregnancy complication/ or exp intrauterine infection/ or exp maternal hypertension/ or exp neoplastic pregnancy complications/ or exp pregnancy diabetes mellitus/ or exp umbilical cord complication/ | 159173 |
| 11 | exp abortion/ | 93259 |
| 12 | prenatal injury/ | 186 |
| 13 | exp puerperal disorder/ or exp maternal disease/ | 54248 |
| 14 | lactation disorder/ | 1265 |
| 15 | exp mastitis/ | 11852 |
| 16 | (matern* adj3 (health* or wellbeing or well-being or wellness or mortality or death* or disease* or infect* or condition* or stress*)).mp. | 103197 |
| 17 | ("pregnancy complication*" or "pregnancy outcome*" or "birth complicat*" or "birth* outcome*").mp. | 164050 |
| 18 | ((prenatal or antenatal or postnatal or perinatal or antepartum or postpartum or obstetric* or pregnan*) adj3 (care or service* or check-up* or checkup* or health* or visit*)).mp. | 141096 |
| 19 | ((pregnan* or gestation*) and (hypertensi* or "high blood pressure*" or "elevated blood pressure*")).mp. | 77557 |
| 20 | (eclamp* or preeclamp* or pre-eclamp*).mp. | 83759 |
| 21 | mother/ or expectant mother/ | 105370 |
| 22 | (pregnan* or mother* or childbearing).mp. | 1325599 |
| 23 | or/1-22 | 1476535 |
| 24 | exp carbon cycle/ or exp carbon footprint/ or exp carbon sequestration/ or exp climate change/ or exp global warming/ or exp desertification/ or exp deglaciation/ or exp drought/ or exp El Nino/ or exp flooding/ or exp greenhouse effect/ or high temperature/ or exp sea level rise/ or exp sea surface temperature/ or exp storm surge/ or severe weather/ or "cold wave (weather)"/ or extreme weather/ or heat wave/ or hurricane/ or "storm (weather)"/ or tornado/ or wildfire/ or ("Arctic amplification" or "Arctic shrinkage" or "carbon footprint" or "carbon offset*" or "carbon sequestration" or "carbon sink" or "carbon sinks" or (chlorofluorocarbon* adj2 (release or concentration* or atmosphere*)) or (climate adj2 (change* or model?ing or predict* or resilience or sensitivity)) or deglaciation or desertification or "natural disaster*" or drought or (earth adj2 warming) or "emissions reduc*" or "emissions trading" or flood* or ((glacial or glacier) adj3 (retreat* or melt*)) or "global radiation" or "global temperature*" or "global warming" or "greenhouse effect" or "greenhouse gas" or "heat wave*" or hurricane* or "ice cap melt*" or "ice mass loss*" or "Kyoto Protocol" or "landslide*" or "land slide*" or "mudslide*" or "mud slide*" or (ozone adj2 hole) or "Paris Accord" or "permafrost melt*" or "polar amplification" or "polar ice melt*" or "sea ice shrinkage" or "sea level rise*" or "sea surface warming" or "storm surge*" or "thermohaline circulation" or ((extreme or severe or destructive) adj2 (temperature* or weather* or storm* or hail or wind* or heat)) or tornado* or typhoon* or wildfire* or "wild fire*").mp. or (("El Nino" or "La Nina").mp. not spanish.lg.) | 230377 |
| 25 | heat shock/ or heat stress/ | 22044 |
| 26 | (heat adj2 (stress* or shock* or cramp* or stroke*)).mp. | 132488 |
| 27 | 24 or 25 or 26 | 355351 |
| 28 | 23 and 27 | 5845 |
| 29 | exp animals/ not exp humans/ | 5186303 |
| 30 | (pollen* or plant* or flora or soil* or agricultur* or "organic matter" or chlorophyll* or crop? or seedling* or seed? or cell? or genus or chromosom* or species or aphid*).ti. | 3254486 |
| 31 | (pig? or rat? or animal* or mouse or mice or cow? or poultry).ti. | 2056435 |
| 32 | 29 or 30 or 31 | 8087857 |
| 33 | 28 not 32 | 3808 |
| 34 | limit 33 to (english language and yr="2013 -Current") | 2739 |
| 35 | limit 34 to conference abstracts | 629 |
| 36 | 34 not 35 | 2110 |

| **Global Health <1910 to 2024 Week 13>** |  |  |
| --- | --- | --- |
|  |  |  |
| 1 | ((matern* or perinatal or pregnan*) adj3 (health* or wellbeing or well-being or wellness or mortality or death* or disease* or infect* or condition* or stress*)).mp. | 48538 |
| 2 | ("pregnancy complication*" or "pregnancy outcome*" or "birth complicat*" or "birth* outcome*").mp. | 25444 |
| 3 | ((prenatal or antenatal or postnatal or perinatal or antepartum or postpartum or obstetric* or pregnan*) adj3 (care or service* or check-up* or checkup* or health* or visit*)).mp. | 28947 |
| 4 | ((pregnan* or gestation*) and (hypertensi* or "high blood pressure*" or "elevated blood pressure*")).mp. | 7066 |
| 5 | (eclamp* or preeclamp* or pre-eclamp*).mp. | 7429 |
| 6 | (pregnan* or mother* or childbearing).mp. | 226759 |
| 7 | or/1-6 | 237535 |
| 8 | ("Arctic amplification" or "Arctic shrinkage" or "carbon footprint" or "carbon offset*" or "carbon sequestration" or "carbon sink" or "carbon sinks" or (chlorofluorocarbon* adj2 (release or concentration* or atmosphere*)) or "climate change*" or deglaciation or desertification or "natural disaster*" or drought or (earth adj2 warming) or El Nino or La Nina or "emissions reduc*" or "emissions trading" or flood* or ((glacial or galcier) adj3 (retreat* or melt*)) or "global radiation" or "global temperature*" or "global warming" or "greenhouse effect" or "greenhouse gas" or "heat wave*" or hurricane* or "ice cap melt*" or "ice mass loss*" or "Kyoto Protocol" or "landslide*" or "land slide*" or "mudslide*" or "mud slide*" or (ozone adj2 hole) or "Paris Accord" or "permafrost melt*" or "polar amplification" or "polar ice melt*" or "sea ice shrinkage" or "sea level rise*" or "sea surface warming" or "storm surge*" or "thermohaline circulation" or ((extreme or severe or destructive) adj2 (temperature* or weather* or storm* or hail or wind* or heat)) or tornado* or typhoon* or wildfire* or "wild fire*").mp. | 42964 |
| 9 | (heat adj2 (stress* or shock* or cramp*)).mp. | 10517 |
| 10 | 8 or 9 | 52580 |
| 11 | 7 and 10 | 1132 |
| 12 | limit 11 to (english language and yr="2013 -Current") | 652 |

| **Database: CINAHL Plus with Full Text via EBSCOhost (1936 - present)** |  |  |
| --- | --- | --- |
| Date of search: January 13, 2024 |  |  |
|  |  |  |
| S1 | (MH "Maternal-Child Health") | 3,864 |
| S2 | (MH "Maternal Mortality") | 6,925 |
| S3 | (MH "Perinatal Death") | 9,291 |
| S4 | (MH "Maternal Health Services+") OR (MH "Postnatal Care+") | 36,434 |
| S5 | (MH "Obstetric Care+") OR (MH "Delivery, Obstetric+") OR (MH "Intrapartum Care+") OR (MH "Vaginal Birth+") OR (MH "Labor, Induced+") | 58,706 |
| S6 | (MH "Pregnancy+") OR (MH "Childbirth+") OR (MH "Pregnancy, Multiple+") OR (MH "Pregnancy Trimesters+") OR (MH "Pregnancy, Unplanned") OR (MH "Pregnancy, Unwanted") OR (MH "Prenatal Exposure Delayed Effects") OR (MH "Prenatal Nutritional Physiology") | 247,273 |
| S7 | (MH "Labor+") | 16,124 |
| S8 | (MH "Pregnancy Outcomes") | 28,070 |
| S9 | (MH "Pregnancy Complications+") OR (MH "Abortion, Spontaneous+") OR (MH "Fetal Diseases+") OR (MH "Labor Complications+") OR (MH "Placenta Diseases+") OR (MH "Pregnancy Complications, Cardiovascular+") OR (MH "Pregnancy Complications, Infectious+") OR (MH "Pregnancy Complications, Neoplastic+") OR (MH "Pregnancy Complications, Psychiatric+") OR (MH "Pregnancy in Diabetes+") OR (MH "Puerperal Disorders+") OR (MH "Obstetric Emergencies") | 110,075 |
| S10 | (matern* N3 (health* or wellbeing or well-being or wellness or mortality or death* or disease* or infect* or condition* or stress*)) | 41,624 |
| S11 | ("pregnancy complication*" or "pregnancy outcome*" or "birth complicat*" or "birth* outcome*") | 63,648 |
| S12 | ((prenatal or antenatal or postnatal or perinatal or antepartum or postpartum or obstetric* or pregnan* or labor) N3 (care or service* or check-up* or checkup* or health* or visit*)) | 80,844 |
| S13 | ((pregnan* or gestation*) and (hypertensi* or "high blood pressure*" or "elevated blood pressure*")) | 13,280 |
| S14 | (eclamp* or preeclamp* or pre-eclamp*) | 17,510 |
| S15 | (MH "Mothers") OR (MH "Expectant Mothers") OR (MH "Multiparas") OR (MH "Primiparas") | 50,709 |
| S16 | (pregnan* or mother* or childbearing) | 364,213 |
| S17 | S1 OR S2 OR S3 OR S4 OR S5 OR S6 OR S7 OR S8 OR S9 OR S10 OR S11 OR S12 OR S13 OR S14 OR S15 OR S16 | 425,215 |
| S18 | (MH "Climate Change+") OR (MH "Sea Level Rise") OR (MH "Greenhouse Effect") OR (MH "Carbon Footprint") OR (MH "Natural Disasters+") OR(MH "Extreme Weather") OR (MH "Wildfires") or ("Arctic amplification" or "Arctic shrinkage" or "carbon footprint" or "carbon offset*" or "carbon sequestration" or "carbon sink" or "carbon sinks" or (chlorofluorocarbon* N2 (release or concentration* or atmosphere*)) or (climate N2 (change* or model* or predict* or resilience or sensitivity)) or deglaciation or desertification or "natural disaster*" or drought or (earth N2 warming) or "emissions reduc*" or "emissions trading" or flood* or ((glacial or glacier) n3 (retreat* or melt*)) or "global radiation" or "global temperature*" or "global warming" or "greenhouse effect" or "greenhouse gas" or "heat wave*" or hurricane* or "ice cap melt*" or "ice mass loss*" or "Kyoto Protocol" or "landslide*" or "land slide*" or "mudslide*" or "mud slide*" or (ozone N2 hole) or "Paris Accord" or "permafrost melt*" or "polar amplification" or "polar ice melt*" or "sea ice shrinkage" or "sea level rise*" or "sea surface warming" or "storm surge*" or "thermohaline circulation" or ((extreme or severe or destructive) N2 (temperature* or weather* or storm* or hail or wind* or heat*)) or tornado* or typhoon* or wildfire* or "wild fire*") | 28,432 |
| S19 | (MH "Heat Stress Disorders+") | 2,581 |
| S20 | (heat N2 (stress* or shock* or cramp*)) | 4,717 |
| S21 | S18 OR S19 OR S20 | 33,738 |
| S22 | S17 AND S21 Limit to Pub Date 2013-2023, English language, Scholarly Peer-Reviewed Journals | 647 |

| **Database: Scopus (1976 - Present)** |  |
| --- | --- |
| Date of search: January 13, 2024 |  |
|  |  |
|  |  |
| ( ( ( TITLE-ABS-KEY ( ( matern* W/3 ( health* OR wellbeing OR well-being OR wellness OR mortality OR death* OR disease* OR infect* OR condition* OR stress* ) ) ) OR TITLE-ABS-KEY ( ( "pregnancy complication*" OR "pregnancy outcome*" OR "birth complicat*" OR "birth* outcome*" ) ) OR TITLE-ABS-KEY ( ( ( prenatal OR antenatal OR postnatal OR perinatal OR antepartum OR postpartum OR obstetric* OR pregnan* ) W/3 ( care OR service* OR check-up* OR checkup* OR health* OR visit* ) ) ) OR TITLE-ABS-KEY ( ( ( pregnan* OR gestation* ) AND ( hypertensi* OR "high blood pressure*" OR "elevated blood pressure*" ) ) ) OR TITLE-ABS-KEY ( ( eclamp* OR preeclamp* OR pre-eclamp* ) ) OR TITLE-ABS-KEY ( ( pregnan* OR mother* OR childbearing ) ) ) ) AND ( TITLE-ABS-KEY ( ( climate OR climatic OR weather ) W/2 ( chang* OR disrupt* OR volati* OR instabilit* OR unstable OR variable OR variability OR vulnerab* ) ) OR TITLE-ABS-KEY ( ( global* OR climate ) W/2 warm* ) OR TITLE-ABS-KEY ( ( extreme OR severe OR destructive ) W/1 ( weather OR heat OR hot* OR temperature* OR storm* ) ) OR TITLE-ABS-KEY ( flood* OR drought* OR hurricane* OR cyclone* OR storm? OR wildfire* OR famine ) ) ) AND NOT ( TITLE ( ( pollen* OR plant* OR flora OR soil* OR agricultur* OR "organic matter" OR chlorophyll* OR crop? OR seedling* OR seed? OR cell? ) ) OR TITLE ( ( pig? OR rat? OR animal* OR mouse OR mice OR cow? OR bull? OR poultry ) ) ) | Maternal health |
| Limit to Pub Date 2013-2023, English Language, Pub Type Articles and Reviews |  |
| Results 2028 |  |
