# Supplementary material for: Impact of climate change on maternal health outcomes: An evidence gap map review
Source: PLOS Glob Public Health. 2024 Aug 19;4(8):e0003540. doi: 10.1371/journal.pgph.0003540 (PMC11332935; doi:10.1371/journal.pgph.0003540)
Supplement: S4 File — (DOCX) [file pgph.0003540.s004.docx]

**S4** **File**. Data Extraction for climate change and maternal health outcomes (EGM)

| **Sr. No.** | **Author name** | **Year** | **Study design** | **Country** | **Sample size** | **Data collection method** | **Age** | **Events/Intervention** | **Outcomes** | **Quality of the study** |
| --- | --- | --- | --- | --- | --- | --- | --- | --- | --- | --- |
| 1 | Abdo et al., 2019 | 2019 | Cross sectional | USA | n=535,895 birth records | Birth records | 18-above 35 | Wildfire | Preterm birth Gestational diabetes Gestational hypertension | High |
| 2 | Abdullah et al., 2019 | 2019 | Qualitative | Bangladesh | n=11 | Interviews | FDGs = 18–50 years, In-depth interviews = 25–60 years | Flood | Maternal morality | High quality |
| 3 | Amjad et al., 2021 | 2021 | Systematic review | Canada | n=10 |  |  | Wildfire | LBW Infant mortality | High quality |
| 4 | Ancheta et al., 2022 | 2022 | Qualitative | Talisay, Batangas |  | Semi-structured interview |  | Disaster | Socio-economic challenges | Moderate |
| 5 | Anwar et al., 2013 | 2013 | Quantitative | Pakistan, Balakot | N=387 | Survey | 15–49 years | Earthquake | Infant mortality; Stillbirth | High |
| 6 | Aryal et al., 2022 | 2022 | Quantitative | Nepal | n=452 | Survey | 15–49 years | Flood | Maternal malnutrition | Moderate |
| 7 | Asamoah et al., 2018 | 2018 | Quantitative | Ghana | n=1136 women | Survey | 15–49 years | Ambient heat temperature | Stillbirths; Miscarriage | High |
| 8 | Auger et al., 2017 | 2017 | Retrospective cohort study | Canada | n=704209 |  | 25–35 years | Ambient heat exposure | Congenital heart defects | High quality |
| 9 | Auger et al., 2014 | 2014 | Time-to-event study design | Canada | n=3660 |  | 20–35 years | Ambient temperature | Preterm birth | Medium |
| 10 | Auger et al., 2017 | 2017 | Quantitative | Canada | Total Pregnancies n= 1 890 711 | Hospital discharge summaries | 25–35 years | Ambient temperature | Preeclampsia | Moderate |
| 11 | Baharav et al., 2023 | 2023 | Review | USA | N/A | N/A |  | Extreme heat | Placental abruption, Preterm birth, Miscarriage, Stillbirth, Neonatal complications, Fetal growth restriction and low birth weight, Congenital birth defects, Diarrheal disease, Vector-borne diseases, Sudden infant death syndrome | Moderate |
| 12 | Barreca and Schaller, 2020 | 2020 | Observational study/Cross sectional | USA | Women and Record | Record | 15–44 years | High ambient temperatures | Preterm birth | Low quality |
| 13 | Wallemacq et al., 2020 | 2020 | Quantitative | Bangladesh | n=4620 | Survey | 15 and 49 years | Floods | Less assess to Maternal and Newborn Healthcare (MNH) utilization | Moderate |
| 14 | Bogan et al., 2019 | 2019 | Quantitative | Turkey | Data records |  |  | Dust storms | Toxemia pregnancy | Moderate |
| 15 | Bonell et al., 2022 | 2022 | Cross sectional | Africa | n=92 |  | 16 years or older | heat stress | Physiological effect of mother and fetus (Strain) | Moderate |
| 16 | Booth et al., 2017 | 2017 | Cross sectional | Canada | n=396 828 women | Records from diabetes database | 30.9 ± 5.4 years | Environmental temperature | Gestational diabetes mellitus | High |
| 17 | Phillips et al., 2020 | 2020 | Qualitative | Canada | n=54 women | Online Interviews | 24–42 years | Wildfire | Psychological effects | High |
| 18 | Brew et el., 2022 | 2022 | Retrospective observational study (Cohort). | Sydney | n = 60054 | Electronically | 25–40 years | Catastrophic bushfire | Low birth weight, Gestational diabetes | Moderate |
| 19 | Brock et al., 2015 | 2015 | Longitudinal observational design | USA | n=171 women | Survey | Average 28.82 years | Floods | Flood related peritraumatic distress | Moderate |
| 20 | Bryson et al., 2021 | 2021 | Qualitative | Uganda | n=24 Batwa and 22 Bakiga women | FGDS | 18 years or above | Sunshine and heat, Extended drought | Low maternal energy, Low birth weight | High |
| 21 | Olah and Frankowska, 2014 | 2014 | Systematic review | Australia | n=7 studies |  |  | Heat stress | Preterm birth | Moderate |
| 22 | Cheng et al., 2020 | 2020 | Observational | China |  | Daily data on PTB | < 35 years > 35 years | Cold temperature | Preterm birth | High |
| 23 | Chersich et al., 2020 | 2020 | Systematic review and random effects meta-analysis |  | n=70 studies |  | 17–41 years | Heatwaves | Preterm birth | High |
| 24 | Christopher et al., 2018 | 2018 | Retrospective, Cross-sectional cohort study | USA |  | National Center for Health Statistics data | 18 and 26 years | Tornadoes | Preterm birth | Moderate |
| 25 | Cox et al., 2016 | 2016 | Cross sectional | Belgium | n=807 835 all births | Centre for Perinatal Epidemiology (SPE) data | < 25 ≥ 35 | Ambient temperature | Preterm delivery | Moderate |
| 26 | Currie and Slater, 2013 | 2013 | Observational study | USA | All Texas births n = 4,193,778, | Database  Texas Department of State Health Services | 25–34 years | Hurricane | Low birth weight | Moderate |
| 27 | Cushing et al., 2022 | 2022 | Retrospective cohort study | USA | Total births n= 198,013 | Texas Department of State Health Services data | Gestational age <20 weeks, Gestational age ≥ 43 | Extreme heat | Preterm birth | Moderate |
| 28 | Davis et al., 2022 | 2022 | Qualitative | Australia | n=43 | Semi-structured interviews | 21–50 years | Bushfires | Physical and psychological impact | High |
| 29 | Dong et al., 2014 | 2014 | Cross sectional design | China | n=344 | Survey | 19–44 years | Earthquake | Iron deficiency anemia | Moderate |
| 30 | Dong et al., 2013 | 2013 | Exploratory and comparative cross-sectional survey | China | n=520 | Questionnaire | 18 ≥30 | Earthquake | Depression | Moderate |
| 31 | Evans et al., 2021 | 2021 | Integrative review | Australia | n= 16 studies |  |  | Wildfire | Low birth weight, Gestational Diabetes Mellitus (GDM) and Gestational hypertension | High |
| 32 | Futterman et al., 2023 | 2023 | Systematic review | USA | n=22 studies | Database | 24–33 years | Earthquake, Hurricane | Depression, Anxiety, PSD | High |
| 33 | Gat et al., 2021 | 2021 | Population-based retrospective cohort study | Israel | n=63,027 births | University Medical Center (SUMC) data | 18–50 years | High temperature | Spontaneous preterm labor | High |
| 34 | Giarratano et al., 2018 | 2018 | Mixed method study | USA | n=402 women | Survey | 18–45 years | Hurricane Katrina | Stress | Moderate |
| 35 | Girardi and Bremer, 2022 | 2022 | Commentary | USA |  |  |  | Heat | Preterm birth (PTB), Low birth weight (LBW) and Stillbirth | High |
| 36 | Hajdu and Gábor Hajdu, 2021 | 2021 | Cross sectional | Hungary |  | Data from the Hungarian Central Statistical Office | 16–44 years | Extreme heat | Pregnancy loss rate | Low quality |
| 37 | Harville et al., 2015 | 2015 | Quantitative | USA | n=308 | Survey | Above 18 years | Hurricane Katrina | Low birthweight | Moderate |
| 38 | Harville et al., 2022 | 2022 | Quantitative | USA |  |  | Average 27.73 | Hurricane | Low birth weight | High |
| 39 | Hawkins et al., 2018 | 2018 | Retrospective cohort study | New Zealand |  | Electronic medical records | 29 Mean age | Earthquake | Preterm birth | Moderate |
| 40 | Neal et al., 2021 | 2021 | Quantitative | California | n=3,002,014 births |  | 28.1 Mean age | Wildfire | Preterm birth | Moderate |
| 41 | Rong He et al., 2016 | 2016 | Quantitative | China | n=838,146 singleton vaginal births | Data from Guangzhou Perinatal Health Care and Delivery Surveillance System (GPHCDSS), | < 20 to > 34 year | High temperature | Preterm birth | Moderate |
| 42 | Siyi He et al., 2017 | 2017 | Case-crossover study design | Canada | n=17,172 women | Data from Study of Hospital Clientele registry, | <25 to ≥35 years | Extreme temperature | Placental abruption | Moderate |
| 43 | Hetherington et al., 2021 | 2021 | Quasi-experimental | Canada | n=18291 |  | 28.9 Mean age | Flood | Preterm birth, Small for gestational age, Preeclampsia, Gestational hypertension, Depression or anxiety | High |
| 44 | Howells et al., 2022 | 2022 | Quantitative | USA | n=37 | Survey | 32.2 Mean age for Married women, 25.8 Mean age for Unmarried | Hurricane Florence | Maternal stress | Moderate |
| 45 | Huang et al., 2021 | 2021 | Case-control design | USA | n=25,328,335  births |  | ≤20 to ≥35 years | Heatwaves | Preterm birth | Moderate |
| 46 | Ilango et al., 2020 | 2020 | Observational study/Cross sectional | California | n = 1,967,300 | data maintained and provided by California Department of Public Health (Birth Data Files 2005–2013) | < 20 to > 35 years | Extreme heat | Preterm birth | High |
| 47 | Jefers et al., 2022 | 2022 | Qualitative | USA | n=18 | Semi-structured interviews | 18-42 | Hurricanes irma and maria | Household/Community, Dimished psychosocial support | High |
| 48 | Jegasothy et al., 2021 | 2021 | Observational study/Cross sectional | Australia | n=916,678 |  | <20 to, ≥35 | Ambient temperature | Preterm birth | High |
| 49 | Khatri et al, 2020 | 2020 | Prospective, Population-based cohort study | Nepal | n=497 | Structured interview | 26.5 (4.8) Mean age | Earthquake | Low birth weight | Moderate |
| 50 | Khatri and fusher, 2019 | 2019 | Systematic review |  |  | Electronic databases |  | Earthquake | Antenatal common mental disorders | High |
| 51 | KOHAN et al., 2016 | 2016 | Qualitative study | Azerbaijan |  | Semi-structured interviews | 15–49 years | Earthquake | Psychological complications | High |
| 52 | Kubota et al., 2018 | 2018 | Quantitative | Japan | n=152 | Questionnaires | 32.1 Mean age | Earthquake | Postpartum depression | Moderate |
| 53 | Kyozuka et al., 2020 | 2020 | Quantitative | Japan | n=8323 women | Survey | 29.7 Mean age | Earthquake | PTB | Moderate |
| 54 | Lai et al., 2015 | 2015 | Quantitative | USA | n=283 mothers |  | 39.20 years | Hurricane | Maternal hurricane exposure and Hurricane-related stressors, Maternal trauma exposure, Maternal perceived social support | Moderate |
| 55 | Whalen., 2020 | 2020 | Qualitative case study | USA | n=8 | Case studies | 20–29 years | Hurricane | Domestic violence and rape | Moderate |
| 56 | Liang et al., 2018 | 2018 | Retrospective observational study | China | n=73,493 | Survey | <20 years to ≥35 years | Earthquake | Stillbirth, PTB, LBW, and SGA | Moderate |
| 57 | Guoao Li et al., 2022 | 2022 | Observational study/Cross sectional | Africa | n=106 382 births | Survey | 15–49 | High temperature | Macrosomia | Moderate |
| 58 | Yanfen Lin et al., 2017 | 2017 | Cross sectional study/Cross sectional |  | n=1931 | In-person interviews | 25-35 years | Ambient temperature | Maternal stress | High |
| 59 | Shanshan Li et al., 2018 | 2018 | Quantitative | Australia | n=289,351 births | Data (Records) | <35 years and >above 35 years | Ambient temperature | Preterm birth, still birth | Moderate |
| 60 | McElroyet al., 2012 | 2012 | Observational study/Cross sectional | USA |  | Questionnaires, Survey | 15–49 years | Extreme heat | Preterm birth, still birth | High |
| 61 | Mendez-Figueroa et al., 2019 | 2019 | Observational study/Cross sectional | USA | n=29,179 deliveries | Survey | 29.7 Mean age | Hurricane Harvey landfall | Maternal morbidity, Neonatal morbidity | High |
| 62 | Mesrkanlou et al., 2022 | 2022 | Cross sectional retrospective descriptive study | Iran | n=550 pre-earthquakes, 450  post-earthquake pregnant women | Data from Ministry of Health for Pregnant Women | 25.82 Mean age | Earthquake | Gestational weight gain, Stillbirth, Term birth | Moderate |
| 63 | Murakami et al., 2023 | 2023 | Observational cohort study | Japan | n=11,403 participants | Questionnaires | ≤29 to ≥35 years | Great east Japan earthquake | Postpartum depressive symptoms | Moderate |
| 64 | MURESAN et al., 2016 | 2016 | Retrospective cohort study, Quantitative | Romania | n=138 cases | Birth record | <20 to > 34 years | Temperature variability | Premature birth | Moderate |
| 65 | Nishigori et al., 2014 | 2014 | Cross sectional study | Japan | n=633 | Questionnaires | <25 to ≥35 | Great east Japan earthquake | Postnatal depression | Moderate |
| 66 | Nowak-Szczepanska et al., 2021 | 2021 | Cross sectional | India | n=597 Aila-exposed women and their offspring (N = 238) Aila-exposed women and their children (N = 138), | Survey | 29.21 Mean age | Natural disaster (Cyclone) | Body mass index (BMI), Mid-Upper Arm Circumference (MUAC) | Low |
| 67 | Olson and Metz, 2022 | 2022 | Review | Canada |  |  |  |  | Effects of the climate crisis on mental health, Prenatal maternal stress leads to adverse pregnancy, newborn, and generational outcomes |  |
| 68 | Palmeiro-Silva et al., 2018 | 2018 | Quantitative | America | n=1966 vaginal deliveries | Birth record | 24-31 years | Earthquake | Increase head circumference, Gestational age | Moderate |
| 69 | Parayiwa et al., 2022 | 2022 | Retrospective cohort study | Australia | Cyclone Yasi (2011): Affected (n=6,587);  Unaffected (n=37,557);  Cyclone Marcia (2015): Affected (n=2,126); Unaffected (n=33,158)  Cyclone Debbie (2017):  Affected (n=6,655); Unaffected (n=15,859) | Birth record | <35 to <35 | Cyclone | Low birthweight, Preterm birth | Moderate |
| 70 | Park et al., 2021 | 2021 | A population-based cohort study | USA | n=2,093,185 births | Hospital administrative data | 28.3 Mean age | Wildfire | Gastroschisis | High |
| 71 | Partash et al., 2022 | 2022 | Systematic review | Iran | n=7 studies | electronic database |  | Flood | Preterm birth, Preeclampsia and Eclampsia, | Moderate |
| 72 | Pomer et al., 2019 | 2019 | Quantitative | Vanuatu | n=926 women in 2015 and 1365 in  2016 | Self-report questionnaire | 32.9 years Mean age | Cyclone | Psychological distress | Moderate |
| 73 | Samuels et al., 2022 | 2022 | Review |  |  | Electronic Databases |  | Ambient heat | Heat exposure and reduced placental blood flow, Oxytocin and prostaglandin release | Moderate |
| 74 | Young Son et al., 2022 | 2022 | Observational cohort study | USA |  | Data from the NC Vital Statistics Reporting System, Department of Health and Human Services, | <20 to ≥35 | Heat exposure | Preterm birth | Moderate |
| 75 | Syed and P. Phillips, 2022 | 2022 | Scoping review |  | n=84 studies | Electronic databases |  | Heat exposure | Preterm birth, Low birthweight, Congenital anomaly, Stillbirth | Moderate |
| 76 | Cabrera et al., 2015 | 2015 | Quantitative | Japan | n=95,069 births | Data from Swedish Medical Birth Register. | 22–42 weeks | Seasonal temperature | Preterm birth | High |
| 77 | Runkle et al., 2022 | 2022 | A retrospective cohort design | USA | n=570,660 women | Hospital-delivery discharge, birth, and maternal mortality records | <19 to >40 | Ambient temperature | Maternal morbidity, Eclampsia and Pregnancy-related hypertension | Moderate |
| 78 | Richards et al., 2022 | 2022 | Matched case–control design | USA | n=140,428 stillbirths |  | 10–19, 20–24, 25–29, 30–34, 34–39, and ≥40 | Heatwaves | Still birth | High |
| 79 | Cabrera et al., 2014 | 2014 | Time-series quasi-Poisson generalized additive models | Spain | Total births n=20,148 | Neonatal Screening Program of the Health Department of Regional Government of Valencia | <25 to >36 | High temperature | Preterm birth | Moderate |
| 80 | Simeone et al., 2023 | 2023 | Quantitative | USA | n=517 women | Survey | 25–34 years | Hurricane | Postpartum depressive symptoms | Moderate |
| 81 | Preston et al., 2020 | 2020 | Systematic review |  | n=16 studies | PubMed and EMBASE databases |  | Temperature (Summer and Winter) | Gestational diabetes mellitus | Moderate |
| 82 | Sajow et al., 2021 | 2021 | Qualitative | Indonesia |  | Interviews | 18-24, and 25-39 years | Volcanic eruption | Maternal and reproductive health care services | Moderate |
| 83 | Ruan et al., 2023 | 2023 | Systematic review and meta-analysis | China | n=34 studies | PubMed, Cochrane, and Embase electronic databases |  | Low ambient temperature | Preterm birth, Low birth weight, still birth |  |
| 84 | Ren et al., 2014 | 2014 | Systematic review | China | n=8 studies | Electronic databases |  | Sichuan earthquake | Depression and Post-traumatic stress disorder | Moderate |
| 85 | Rammah et al., 2019 | 2019 | Case-crossover study | USA | n=709 stillbirth | Fetal Health Record | <20 to ≥ 40 | High temperature | Stillbirth | High |
| 86 | Veenema et al., 2023 | 2023 | Systematic review | USA | n=19 studies | Web of Science and PubMed |  | Extreme temperatures; Air pollution; and Natural disasters | Preterm birth, Stillbirth, Low birth weight | Moderate |
| 87 | Sun et al., 2019 | 2019 | Retrospective observational study | USA | n=total births 2,973,909 | Data Record | <25 to ≥35 | Extreme heat and cold | Preterm birth | High |
| 88 | Zhang et al., 2017 | 2017 | Systematic review | China | n=36 studies | Electronic databases including PubMed, Medline, Scopus, Web of Science and Google Scholar |  | Extreme heat and cold | PTB, LBW, and Stillbirth | Moderate |
| 89 | Saulnier and Brolin, 2015 | 2015 | Systematic review | USA | n=47 studies | Electronic databasesa |  | Extreme cold weather events, Extreme heat events, Floods or storms, Chemical exposure, Nuclear radiation, Famine, War | Birth and reproductive outcomes, Mental health or illness, Mortality, Metabolic outcomes, Cardiovascular disease, Cancer, | High |
| 90 | Poursafa et al., 2015 | 2015 | Systematic review | Iran | n=15 studies | Electronic databases (ISI, Web of Knowledge, PubMed, Scopus, and Google Scholar) |  | Climate change (Winter, Summer) | Eclampsia, Preeclampsia, Cataract, LBW, PTB, Hypertension | High |
| 91 | Zhang et al., 2022 | 2022 | Quantitative | Australia |  | Birth record | <20 to ˃ 40 | Daily wildfire-specific PM2.5 | Preterm birth, Low birth weight | High |
| 92 | Schifan et al., 2015 | 2015 | Observational cohort study | Rome | n=Barcelona = 27,255; Rome = 78,633 | Certificate of Delivery Care Registry, Rome and Birth Registry of the Catalan Institute of Statistics, Barcelona | <30 to >37 | Extreme temperature and air pollutants | Preterm birth | Moderate |
| 93 | Wang et al., 2020 | 2020 | Observational cohort study | China | n=1,281,859 singleton pregnancies |  | 18–25, 26–30, 31–35, 36–45 | High temperature | Preterm birth | High |
| 94 | Zhang et al.,2021 | 2021 | Observational study/Cross sectional | China | n=5,421 pregnant women |  | 13–50 years | Extreme low temperature, Extreme high temperature | Gestational diabetes mellitus | High |
| 95 | Wang et al., 2013 | 2013 | An ecological study | Australia | n=50 848 births | Data Collections Unit (DCU) of the Queensland Health Statistics  Centre. | <20 to >34 years | Heatwaves | Spontaneous preterm births | Moderate |
| 96 | Ren et al., 2022 | 2022 | Prospective population-based cohort | China | n=210,798 singleton live births |  | 34.0 Mean age | Extreme temperature | Preterm birth | Moderate |
| 97 | Wang et al.., 2017 | 2017 | Observational quantitative /Cross sectional | Australia | n=277,133 singleton births | Data Collections Unit of Queensland Health | <20 to ≥35 | Heatwaves | Preterm birth | High |
| 98 | Komiya et al., 2015 | 2015 | Observational Study | Japan | n=9321 | Survey, Questionnaires | 30.1 Mean age | Great east Japan earthquake | Depressive symptoms, Preterm birth, still birth and miscarriage | Moderate |
| 99 | Kanner et al., 2020 | 2020 | Retrospective cohort study | USA | n=498 | Electronic medical records | <19 to <35 | Extreme cold | Still birth | Low |
| 100 | Bennett et al., 2019 | 2019 | Case study | Bangladesh | n=370 women | Structured interview | 15–49 years | Flood | Spontaneous abortion and a lack of treatment | Moderate |
| 101 | Severson et al., 2023 | 2023 | A qualitative secondary analysis | USA | n=50 | Questionnaires | 18-40 | Wildfire and hurricane | Psychological symptoms | High quality |
| 102 | Silva-Suarez et al., 2021 | 2021 | Qualitative study | USA | n=10 women | In-depth interviews | 30 Mean age | Hurricane | Psychological symptoms | High |
| 103 | Song et al., 2019 | 2019 | A quasi-Poisson generalized additive model | China |  | Data record | < 35, and >35 years | Extreme heat | PROM | Moderate |
| 104 | Sun et al., 2023 | 2023 | Observational cohort study/Cross sectional | China | n=105063 | Data from the CBCS | 29.60 Mean age | Ambient temperature | Hypertensive disorder in pregnancy | Moderate |
| 105 | Tanoue et al., 2021 | 2021 | Observational cohort study/Cross sectional | Japan | n=76152 women |  | ≤24 to ≥35 | Great east Japan earthquake | Psychological distress | High |
| 106 | Yoshii et al., 2014 | 2014 | Qualitative | Japan | n=259 | Survey | 33.02 ± 4.79 Mean age | Great east Japan earthquake | Anxiety |  |
| 107 | Zhao et al., 2022 | 2022 | Case–control study | China | n=3006 women | Data from Nanjing Maternity and Child Health Care Hospital | 30.49 ± 5.35 Mean age | High temperature | Spontaneous abortion | Low quality |
| 108 | Zhou et al., 2012 | 2012 | Quantitative | China | n=1,231,715 medical records | Birth records | <20 to ≥35 | Ambient temperature (Extreme heat) | Preterm birth | Moderate |
| 109 | Basagaña eta l., 2021 | 2021 | Retrospective cohort study | Israel | n=Singleton term births 624,940 | Used data from the registry | ≤20 to ≥41 | Low and high ambient temperatures | Lower mean birth weight, Reduced fetal growth | Moderate |
| 110 | Chene et al., 2023 | 2023 | Retrospective cohort study | China | n=56,905 singleton pregnant women |  | 18–25, 26–30, 31–35, 36–40, 41–45, > 45 years | Ambient ozone (O3) and temperature | PTB, LBW, SGA, LGA |  |
| 111 | Chen et al., 2020 | 2020 | Case study | China | n=864,757 live singleton births |  | 25.243 Mean age | Heatwaves and cold spells | Low birth weight | Moderate |
| 112 | Cil and Cameron, 2017 | 2017 | Observational study/Cross sectional | USA |  |  | 29–34, and 35 and over | Heat waves | Pregnancy-associated hypertension, Uterine bleeding during pregnancy, Eclampsia, Incompetent cervix | High |
| 113 | Cil and Kim, 2022 | 2022 | Observational study/Cross sectional | USA |  |  | <18 to ≥35 | Extreme temperature | Preterm births, Low birth weight | Moderate |
| 114 | Demirchyan et al., 2014 | 2014 | Case-control study | Armenia | n=146 |  | 31.4 (5.5) Mean age | Earthquake | Post-partum depression | High |
| 115 | FUJIMORI et al., 2014 | 2014 | Survey | Japan | n=8602 women | Questionnaires and Survey | 30.1 Mean age | Earthquake | Spontaneous abortions, Stillbirth, Preterm birth | Moderate |
| 116 | Grabich et al., 2016 | 2016 | Observational study/Cross sectional | Florida | n=342,942 singleton births | Birth data | <18  18–25  25–35  35–45 | Hurricane | Preterm deli every | Moderate |
| 117 | Hajdu and Gábor Hajdu, 2021 | 2021 | Observational study/Cross sectional | Hungary | n=590,872 pregnancy losses | Data | 15–44 years | High temperature | Risk of pregnancy loss | High |
| 118 | Sandie Ha et al., 2016 | 2016 | Retrospective cohort study | USA | n=992 stillbirths and 222,383 No stillbirth | Data from the Air Quality and Reproductive Health study | <20 to ≥35 years | Ambient temperature (Heat and Cold) | Stillbirth | High |
| 119 | Hilmert et al., 2016 | 2016 | Quantitative study | Dakota | n=136 | Questionnaires and Survey | 18-35 or above | Flood | Low birth weight, Neonates due to reduced fetal growth | Moderate |
| 120 | Jiao et al., 2023 | 2023 | Retrospective cohort study | USA | n=190767 | Electronic health record (EHR) | <25 to ≥35 years | Extreme heat | Premature rupture of membrane | Moderate |
| 121 | Requia et al., 2022 | 2022 | Case-crossover study | Brazil | n=190,911 preterm births | Data from Ministry of Health in Brazil | 18–45 | Wildfire smoke | Preterm birth | High |
| 122 | Savitz and Hu, 2021 | 2021 | Case-crossover design | USA | n=1876 stillbirths | Electronic health records data |  | Ambient heat | Stillbirth | Moderate |
| 123 | Sun et al., 2020 | 2020 | Observational study/Cross sectional | USA | n=19,529,74 | Records from the Centers for Disease Control and Prevention (CDC)’s National Center for Health Statistics, US | <30 and >30 | Cyclones | Preterm birth | Moderate |
| 124 | Bahmanjanbeh et al., 2016 | 2016 | Descriptive study | Azerbaijan | n=44,265 | Secondary data, Census method | 15-49 | Earthquake | Decrease of reproductive health | Moderate |
| 125 | Sandie Ha et al., 2017 | 2017 | Case-crossover analyses, Observational cohort study | USA | n=15,381 | Record | <20 to ≥35 | Temperature | Premature rupture of membrane | Moderate |
| 126 | Howells et al., 2020 | 2020 | Observational study/Cross sectional | North Carolina | n=38 in person women  45 online interviews | In-person interviews, Online questionnaire | Over 18 years | Hurricane | Psychological distress |  |
| 127 | Jegasothy et al., 2021 | 2021 | Review | Malaysia |  |  |  | High Temperature | Temperature change and reproductive health; Temperature change and male reproductive functions; Temperature change in female reproduction and pregnancy outcomes; Temperature and reproductive hormones; Haze, air pollution and its impact on fertility; Heavy rainfall, flood and its impact  on fertility; Climate change, infectious diseases and infertility | Moderate |
| 128 | Khatri et al., 2018 | 2018 | Cross-sectional study | Nepal | n=497 women | Structured fixed response option interviews | < 20 to>31 | Earthquake | Common Mental Disorders (CMDs) | High |
| 129 | Kyozuka et al., 2020 | 2020 | Cross sectional | Iran | n=8323 women | Survey | 31.3 Mean age | Earthquake | Hypertensive disorder of pregnancy | Moderate |
| 130 | Previdi et al., 2022 | 2022 | Qualitative | Puerto Rico | n=375 women | Questionnaire | 27 Median age | Hurricanes | Pregnancy and birth challenges, Lack of access to basic services | High |
| 131 | Grecan et al., 2022 | 2022 | Cross sectional | Florida | n=534 | Online survey | <35 years | Evacuation and Hurricane | Emotional distress | Moderate |
| 132 | Kushnick et al., 2021 | 2021 | Retrospective matched cohort-study design | Indonesia | n=97 | Structured interviews | 30.4 Mean age | Volcano | Preterm birth | High |
| 133 | Melody et al., 2019 | 2019 | Systematic review |  | n=11 | Electronic databases |  | Extreme heat | Fetal growth restriction |  |
