# Supplementary material for: Impact of climate change on maternal health outcomes: An evidence gap map review
Source: PLOS Glob Public Health. 2024 Aug 19;4(8):e0003540. doi: 10.1371/journal.pgph.0003540 (PMC11332935; doi:10.1371/journal.pgph.0003540)
Supplement: Attachment — Submitted filename: Rebuttal letter.docx [file pgph.0003540.s005.docx]

Overall Comments:

Dear Editor and reviewers, thank you so much for providing valuable suggestions to improve the quality of our paper. We really appreciate it, and we have addressed your comments. Please find the details below.

Journal Requirements:

1. Please review your reference list to ensure that it is complete and correct. If you have cited papers that have been retracted, please include the rationale for doing so in the manuscript text or remove these references and replace them with relevant current references. Any changes to the reference list should be mentioned in the rebuttal letter that accompanies your revised manuscript. If you need to cite a retracted article, indicate the article’s retracted status in the References list and also include a citation and full reference for the retraction notice.

**Thanks for your comments. We have addressed it.**

2. Please amend your detailed Financial Disclosure statement. This is published with the article. It must therefore be completed in full sentences and contain the exact wording you wish to be published.

**Thank you for your comment. This concern has been addressed.**

a. State the initials, alongside each funding source, of each author to receive each grant.

**Thank you for your suggestions. This comment has been addressed.**

b. State what role the funders took in the study. If the funders had no role in your study, please state: “The funders had no role in study design, data collection and analysis, decision to publish, or preparation of the manuscript.”

**Thanks for your comment. This has been added in the manuscript.**

c. If any authors received a salary from any of your funders, please state which authors and which funders.

If you did not receive any funding for this study, please simply state: “The authors received no specific funding for this work.”

**We have addressed this comment.**

3. We have noticed that you have uploaded Supporting Information files, but you have not included a list of legends. Please add a full list of legends for your Supporting Information files after the references list.

**Thanks for your comment. We have moved it after the references.**

4. In the online submission form, you indicated that "Data will be provided on formal request to the corresponding author".

All PLOS journals now require all data underlying the findings described in their manuscript to be freely available to other researchers, either 
1. In a public repository, 
2. Within the manuscript itself, or 
3. Uploaded as supplementary information.

Thanks for the updates. We have addressed it in the manuscript.

Additional Editor Comments (if provided):

This is a well-written manuscript addressing an important global health issue. Authors should add some descriptive graphs (bar charts, for example) to highlight where evidence is available, and in doing so, also highlight where it is not. I believe that Figure 3 alone is not enough to communicate the results.
Please address the comments from the two reviewers.

Thank you for bringing this to our attention. We have provided a QR code for the link to the map, which shows where evidence is available. It also highlights where this research has been carried out (Countries). To show the gaps in evidence we used figure 3 and also mentioned it in the findings section.

Introduction:
The introduction is clear and sets the context well. It might be useful to present the connections between climate change and mental health in a more structured manner, such as categorising physiological risks, social/economic risks/vulnerability, and indirect risks/harms.

**Thank you for your comments. We have revised it as per your suggestions.**

Line 120: Please clarify if this refers to maternal deaths, and how conservative this estimate is, considering the potential for ambitious adaptation.
The introduction could be edited down slightly with additional structure to aid readability.

**Thank you! This comment has been addressed.**

Methods:
The methods are appropriate and well-described.
It might be beneficial to illustrate the search strategy in the main text rather than just in the supplementary materials.

**Thank you for your comments. We have added these details in the manuscript.**
It would be interesting to see the proportion of grey literature in the identified papers, as I had assumed this would be significant. (We have addressed this comment in the Figure 1)

Findings:

It could be beneficial to compare the trend in publications to the wider explosion in papers/publications.
Figure 5 appears to be misformatted or unclear (e.g., Africa appearing west of South America). I suggest either a histogram or a flat projection with shading to improve clarity. Currently, it slightly distracts from a core finding.
Is there any scope to look beyond the crude counts? It could be useful to disaggregate by the type of paper.

Thank you for your suggestions. We have revised the figure 5 as per your suggestions.

At times, the end of the findings section strays into discussion/implications.

**Thank you for your comment. We have made changes to it.**

Discussion:

Line 261: "Moreover" might not be the best word choice here as it introduces a different and important point.

**Thank you! This comment has been addressed.**

Line 275: It seems to refer to a relative abundance, not an absolute one; it is worth clarifying this.

**Thank you we have made changes to this.**

At times, the language could be clearer and more precise (e.g., Line 290).
The implications section is a little generic. It might be helpful to propose a set of specific recommendations or ideas for consideration.

**Thank you for your comment. We have made changes to this.**

Figures:

Figure 1:

The image resolution is poor.
It would be helpful to disaggregate by the type of study (e.g., grey literature vs peer-reviewed).

**Thank you ! We have revised it per your suggestions.**

Figure 2:

It is challenging to effectively capture elements like the Evidence Map, but the QR code is a good idea.
It could be nice to illustrate a couple of use cases for the map, showing how the data can be utilised.

**Thank you! We have mentioned some details regarding this in the manuscript.**

Figure 3:

The figure could be more visually engaging, possibly by illustrating the points with icons and using more colour. A redrawn version could be helpful to the reader.

**Thank you for your comments! We have revised it accordingly.**

Figure 5:

As previously discussed, the current projection is not appropriate. I suggest using a flat projection with coloured shading.

**Thank you for your comments! We have revised it as per your suggestions.**

Supporting Information:
I have not reviewed the supporting information
